# Sphingolipid Domains in the Plasma Membranes of Fibroblasts Are Not Enriched with Cholesterol*[Fn FN2]

**DOI:** 10.1074/jbc.M113.473207

**Published:** 2013-04-22

**Authors:** Jessica F. Frisz, Haley A. Klitzing, Kaiyan Lou, Ian D. Hutcheon, Peter K. Weber, Joshua Zimmerberg, Mary L. Kraft

**Affiliations:** From the Departments of ‡ Chemistry and; § Chemical and Biomolecular Engineering, University of Illinois, Urbana, Illinois 61801,; the ¶ Glenn T. Seaborg Institute, Lawrence Livermore National Laboratory, Livermore, California 94551, and; the ‖ Program in Physical Biology, Eunice Kennedy Shriver NICHD, National Institutes of Health, Bethesda, Maryland 20892

**Keywords:** Cholesterol, Isotopic Tracers, Lipid Raft, Mass Spectrometry (MS), Membrane Structure, Plasma Membrane, Sphingolipid

## Abstract

The plasma membranes of mammalian cells are widely expected to contain domains that are enriched with cholesterol and sphingolipids. In this work, we have used high-resolution secondary ion mass spectrometry to directly map the distributions of isotope-labeled cholesterol and sphingolipids in the plasma membranes of intact fibroblast cells. Although acute cholesterol depletion reduced sphingolipid domain abundance, cholesterol was evenly distributed throughout the plasma membrane and was not enriched within the sphingolipid domains. Thus, we rule out favorable cholesterol-sphingolipid interactions as dictating plasma membrane organization in fibroblast cells. Because the sphingolipid domains are disrupted by drugs that depolymerize the cells actin cytoskeleton, cholesterol must instead affect the sphingolipid organization via an indirect mechanism that involves the cytoskeleton.

## Teaser

**Background:** Although cholesterol abundance affects cell function, its distribution within the plasma membrane is not established.

**Results:** Cholesterol is uniformly distributed throughout the plasma membrane and is not enriched within sphingolipid domains.

**Conclusion:** Sphingolipid organization in the plasma membrane is not dictated by direct cholesterol-sphingolipid interactions.

**Significance:** Cholesterol abundance affects sphingolipid organization in the plasma membrane via an indirect mechanism.

## Introduction

Although cholesterol concentration is known to vary between different organelles (1), the cholesterol distribution in the plasma membrane is the subject of debate (2). According to one hypothesis, the plasma membrane contains small (<200 nm in diameter) and dynamic domains that are enriched with cholesterol and sphingolipids (3, 4). These domains, which are referred to as lipid rafts, are postulated to result from favorable interactions between cholesterol, sphingolipids, and select membrane proteins (3, 4). Although the cholesterol-dependent biophysical behaviors of sphingolipids have been characterized (5), until recently, the distributions of most lipids in the plasma membrane could not be directly imaged without using potentially perturbing labels (*i.e.* fluorophores).

A high-resolution secondary ion mass spectrometry (SIMS)^2^ technique that does not alter biomolecule distribution in membranes or cellular compartments (6–11) has enabled visualizing the organizations of rare isotope-labeled lipids in the plasma membrane by mapping their distinctive isotope enrichments (10–12). In a recent report, we used high-resolution SIMS, which was performed on a Cameca NanoSIMS 50, to image the distributions of metabolically incorporated ^15^N-sphingolipids in the plasma membranes of intact cells. Our study focused on a mouse fibroblast cell line that stably expressed influenza hemagglutinin (clone 15) because the hypothesis that the micrometer scale clusters of hemagglutinin in their plasma membranes were associated with lipid rafts (13–15) suggested that these cells had sphingolipid domains that could be detected by a NanoSIMS. High-resolution SIMS imaging revealed micrometer scale patches of sphingolipid microdomains in the plasma membranes of the clone 15 cells (11). By comparing this sphingolipid organization to those exhibited by hemagglutinin-free mouse fibroblast cells (NIH 3T3, parent line from which clone 15 was derived) or induced by drugs, we probed the mechanisms of plasma membrane organization. The sphingolipid domains were strongly perturbed by disruption of the cytoskeleton, moderately altered by reductions in cellular cholesterol, and insensitive to the presence of hemagglutinin in the plasma membrane (11). These results indicate that the cytoskeleton and its associated proteins organize the sphingolipids in the plasma membrane. However, neither the mechanism by which cholesterol abundance modulates sphingolipid organization nor the precise cholesterol distribution in the plasma membrane was elucidated.

We have used high-resolution SIMS to image the ^18^O-cholesterol with respect to the ^15^N-sphingolipids in the plasma membranes of fibroblast cells. We assessed whether the ^15^N-sphingolipid domains were enriched with ^18^O-cholesterol, as predicted by the long standing hypothesis that favorable cholesterol-sphingolipid interactions drive the preferential association of these components in membranes (3, 16). We also characterized the effects of acute cholesterol depletion on cholesterol and sphingolipid distribution in the plasma membrane.

## EXPERIMENTAL PROCEDURES

### 

#### 

##### Materials

The clone 15 cell line was obtained by transfecting NIH 3T3 mouse fibroblast cells with a DNA plasmid for hemagglutinin from the 1957 pandemic Japan strain of influenza and selecting for stably transfected cells with standard techniques. Fatty acid-free BSA and other cell culture materials were obtained from Sigma. High-glucose DMEM was prepared by the Cell Media Facility in the School of Chemical Sciences at the University of Illinois. Poly-l-lysine and chemical preservation reagents were purchased from Electron Microscopy Sciences. Methyl-β-cyclodextrin (mβCD) was from Acros Organics. The ^15^N-sphingolipid precursors, ^15^N-sphingosine and ^15^N-sphinganine, were synthesized from ^15^N-serine (Cambridge Isotope Laboratories) using reported methods (17, 18). ^18^O-Cholesterol was synthesized from *i*-cholesteryl methyl ether (Sigma) and ^18^O-water (Olinax, Inc.) as reported previously (19).

##### Metabolic Labeling

Cells were cultured in high-glucose DMEM with 10% calf serum, 104 units/ml penicillin G, 10 mg/ml streptomycin, 3.2 μm of ^15^N-sphingolipid precursors, and 20 μm ethanolamine at 37 °C and 5% CO_2_. After 2 days, additional ^15^N-sphingolipids and ethanolamine were added to produce concentrations of 3.2 μm and 20 μm, respectively, in the culture. After 3 days, the cells were passaged into DMEM supplemented with 1% (v/v) calf serum (Hyclone), 10% (v/v) lipid-reduced FBS (Hyclone), 3.2 μm ^15^N-sphingolipid precursors, 50 μm ^18^O-cholesterol (2:5 mass ratio of ^18^O-cholesterol:fatty acid-free BSA), and 20 μm ethanolamine. On day 4, ^15^N-sphingolipid precursors, ^18^O-cholesterol, and ethanolamine were added to produce concentrations of 3.2, 50, and 20 μm, respectively. On day 5, the cells were passaged into dishes containing 5-mm by 5-mm silicon substrates (Ted Pella) that were coated with poly-l-lysine and were incubated at 37 °C and 5% CO_2_ until day 6.

##### Cell Preservation

The cells growing on the silicon substrates were removed from the culture dish and preserved as reported previously (10). Briefly, the substrates with adherent cells were rinsed twice with PBS (without Ca^2+^ or Mg^2+^), twice with Hendry's phosphate buffer, and fixed for 30 min in 4% glutaraldehyde in Hendry's phosphate buffer. Substrates were then rinsed once for 5 min in Hendry's phosphate buffer, twice for 5 min in triple-distilled water, and post-fixed for 15 min in 0.4% osmium tetroxide in water. Finally, the cells were rinsed in triple-distilled water for 15 min and air-dried.

##### Cholesterol Depletion

Culture dishes containing metabolically labeled cells and substrates with adherent cells were rinsed with 10 ml of PBS (without Ca^2+^ or Mg^2+^), incubated with 15 ml of 10 mm mβCD in DMEM for 15 min at 37 °C, and rinsed with 10 ml of PBS. The cells attached to the silicon substrates were preserved using the procedure described above. The lipids were extracted from the cells remaining on the culture dish using previously reported methods (11), and the cholesterol to phospholipid ratio in the lipid extract was determined with the Amplex® Red Cholesterol and Phospholipase D Assay Kits (Invitrogen) as described previously (7).

##### Assessment of Isotope Incorporation

The cells that were adhered to the culture dish were used to assess isotope incorporation. The lipids were extracted from the cells as reported previously (11). Nitrogen-15 incorporation into the cellular sphingomyelin was measured as reported (10). The fraction of the total cellular cholesterol that contained the oxygen-18 isotope was determined by GC-MS using an Agilent 7890 gas chromatograph equipped with a HP-5m column (30 m, inner diameter × 0.25 mm, 0.25-μm film thickness) capillary column (Agilent Technologies, Inc.), an Agilent 5975C mass selective detector, and HP 7683B (Agilent Technologies, Inc.) autosampler. The signal intensities at *m*/*z* 386 and 388 were used to detect ^16^O-cholesterol and ^18^O-cholesterol, respectively.

##### Low Voltage SEM

Samples were imaged on a Hitachi S4800 high resolution SEM at 1 keV and 8-mm working distance.

##### SIMS Analysis

Samples were coated with 3 nm of iridium using a Cressington 208HR high-resolution sputter coater with a MTM-20 thickness controller. Prior studies by ourselves and others confirm that this thin metal coating does not alter the lipid distribution at the cell surface (11, 20). SIMS was performed with the NanoSIMS 50 (Cameca, France) at Lawrence Livermore National Laboratory (Livermore, CA). 15 × 15 μm regions were analyzed using a 0.077 pA, 15 keV ^133^Cs^+^ primary ion beam with a ∼70-nm spot size. Eight replicate scans of 512 × 512 pixels (pixel size = 29 × 29 nm^2^) were acquired with a dwell time of 1 ms/pixel, resulting in a primary ion dose of 4.5 × 10^14^ ions/cm^2^. The ^12^C^14^N^−^, ^12^C^15^N^−^, ^16^O^−^, and ^18^O^−^ secondary ions were collected with a mass resolving power of ∼6700. A sputter depth of 1.8 nm was calculated as described (21) using the sputtering rate of 2.5 nm·μm^2^/pA·s determined on other biological samples (21), a primary ion beam current of 0.077 pA, and a sputter time of 2097 s. This shallow sputtering depth ensured that the vast majority of the secondary ions were collected from the plasma membrane and not the underlying cytoplasm (10). For each cell, 1–11 15 × 15 μm regions were imaged for comparison of the ^18^O-enrichment in the ^15^N-sphingolipid domain and nondomain regions.

##### Image Analysis

L'image software (L. R. Nittler, Carnegie Institution of Washington) run on the PV-Wave platform (Visual Numerics, Inc.) was used to determine the primary ion beam diameter (70 nm), generate isotope enrichment images, define regions of interest, and export quantitative data from the regions of interest (11). Quantitative ^15^N- and ^18^O-enrichment images were constructed by taking the ratio of the rare isotope-labeled secondary ion counts and the corresponding abundant secondary ion counts at each pixel (^12^C^15^N^−^/^12^C^14^N^−^ and ^18^O^−^/^16^O^−^, respectively). This ratio removed signal intensity variations from concentration-independent factors, such as topography and matrix effects, producing a ratio that is proportional to the abundance of the isotope-labeled species (6, 10, 11, 22). A 3 × 3-pixel boxcar smoothing algorithm was applied to minimize random noise, producing images with 87-nm lateral resolution. Isotope enrichment factors, which are relative measures of the amount of ^15^N-sphingolipids or ^18^O-cholesterol compared with an unlabeled cell, were calculated by dividing the ^12^C^15^N^−^/^12^C^14^N^−^ or ^18^O^−^/^16^O^−^ ratio by the standard abundance ratio (0.00367 and 0.0020052, respectively). The MATLAB statistics toolbox was used to determine the statistically significant thresholds for ^15^N-enrichment and to perform Kolmogorov-Smirnov tests for statistically significant differences in the ^18^O-enrichment in the ^15^N-sphingolipid domains.

## RESULTS

### 

#### 

##### Cholesterol Is Evenly Distributed in the Plasma Membranes of Clone 15 Cells

**FIGURE 1.**
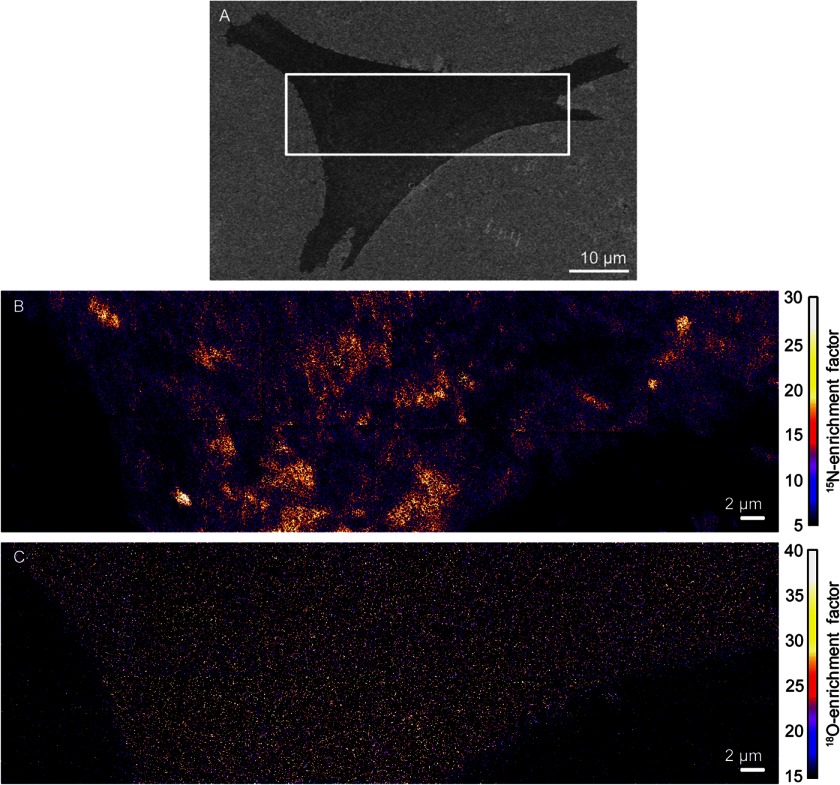
**SEM and high-resolution SIMS images of a representative clone 15 fibroblast cell.** *A*, SEM image of a clone 15 cell. The approximate location that was analyzed with high-resolution SIMS is outlined. *B*, the distribution of metabolically incorporated ^15^N-sphingolipids in the plasma membrane of the clone 15 cell was imaged by detecting the sphingolipid-specific ^15^N-enrichment with high-resolution SIMS. *Orange* and *yellow* regions represent plasma membrane domains that are enriched with ^15^N-sphingolipids. *C*, mosaic of ^18^O-enrichment images acquired with high-resolution SIMS shows the metabolically incorporated ^18^O-cholesterol is relatively uniformly distributed in the plasma membrane.

We studied clone 15 cells that had been metabolically labeled, such that ∼90% of the cellular sphingolipids contained one nitrogen-15 isotope, and ∼60% of the cellular cholesterol contained one oxygen-18 isotope. After chemically fixing the cells with a method that does not alter the lipid distribution in the membrane (11), cells with normal morphologies, as assessed with low-voltage SEM (Fig. 1*A*), were analyzed with high-resolution SIMS. Plasma membrane domains with elevated ^15^N-enrichment are visible in the mosaic of ^15^N-enrichment images of the representative clone 15 cell (Fig. 1*B*). Our previous report confirmed that the ^15^N-enriched domains are not artifacts caused by sample preparation, cell topography, SIMS analysis, or the detection of intracellular membranes (11). For the representative clone 15 cell (Fig. 1*B*), ^15^N-enrichment factors greater than 16.3 were statistically significant elevations that signify ^15^N-sphingolipid-enriched domains (mean (μ) ^15^N-enrichment factor for domain-free regions = 7.5; 1 S.D. = 4.4). Surprisingly, cholesterol-enriched domains are not visible in the ^18^O-enrichment images. Instead, the ^18^O-cholesterol appears to be relatively evenly distributed in the plasma membrane (Fig. 1*C*). No difference in the ^18^O-cholesterol abundance within the sphingolipid domain and non-domain regions was detected by visual inspection or a Kolmogorov-Smirnov statistical test (*p* = 0.96). Similar ^15^N-sphingolipid and ^18^O-cholesterol distributions were observed in the membranes of the four other clone 15 cells we examined (*i.e.* supplemental Fig. S1), and Kolmogorov-Smirnov tests confirmed that their ^15^N-sphingolipid domains were not enriched with ^18^O-cholesterol (*p* = 0.80, 0.54, 0.49, and 0.60; supplemental Table S1).

##### Hemagglutinin Does Not Affect the Cholesterol and Sphingolipid Distribution

**FIGURE 2.**
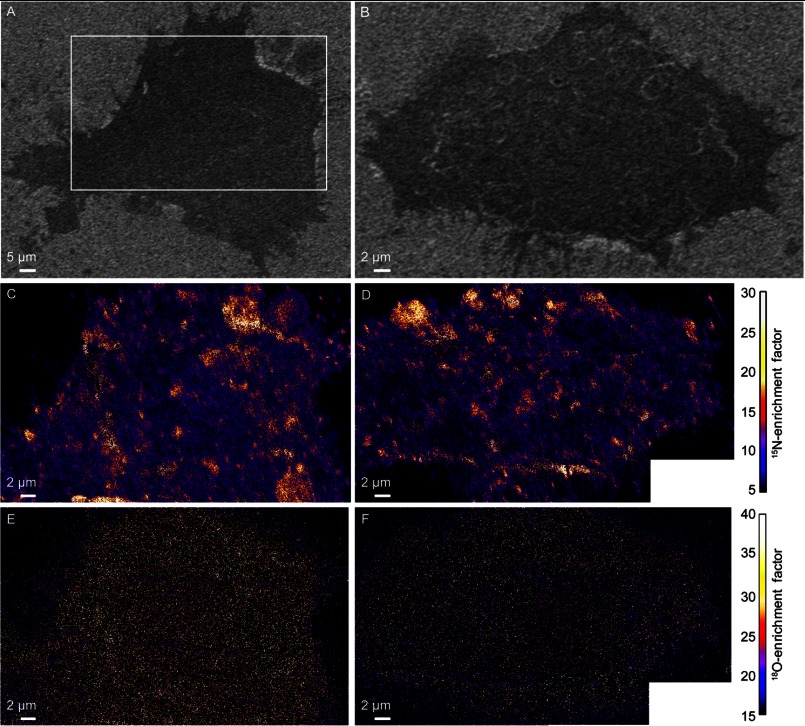
**SEM and SIMS images of two NIH 3T3 mouse fibroblast cells that did not express the influenza membrane protein, hemagglutinin.** *A* and *B*, SEM images show cell morphology. The region outlined in *A* shows the approximate location that was analyzed with high-resolution SIMS. *C* and *D*, mosaics of ^15^N-enrichment SIMS images were acquired on each metabolically labeled cell shown in *A* and *B*, respectively. Domains enriched with ^15^N-sphingolipids (*orange*, *yellow*, and *white* areas) are present in the plasma membrane. *E* and *F*, mosaics of ^18^O-enrichment SIMS images show the distribution of metabolically incorporated ^18^O-cholesterol in the plasma membrane.

We also analyzed mouse fibroblast cells that did not express hemagglutinin (NIH 3T3 cells, parent line from which clone 15 was derived). Plasma membrane domains enriched with ^15^N-sphingolipids were detected on the four hemagglutinin-free NIH 3T3 fibroblasts we examined (Fig. 2, *C* and *D*, and data not shown), whereas the ^18^O-cholesterol appeared to be relatively uniformly distributed in the plasma membrane (Fig. 2, *E* and *F*, and data not shown). Kolmogorov-Smirnov test of the ^18^O-enrichment revealed a small (5%) but statistically significant elevation in ^18^O-cholesterol within the ^15^N-sphingolipid domains (*p* = 0.03, supplemental Table S1) on the cell in Fig. 2*E*. Based on a 0.51/1 cholesterol to phospholipid ratio (mol/mol) in the plasma membrane (23), this 5% elevation corresponds to a <2 mol% increase in cholesterol within the sphingolipid domains. This increase is much smaller than that predicted by phase diagrams for vesicles composed of cholesterol, *N*-palmitoyl sphingomyelin, and an abundant cellular phosphatidylcholine (16:0–18:1 phosphatidylcholine) at 37 °C (24). However, the absence of a significant increase in cholesterol within the sphingolipid domains is consistent with the recent report that cholesterol co-localizes with the ganglioside, GM1, but not sphingomyelin, in model lipid membranes (25). No significant differences in the ^18^O-enrichments in the domain and non-domain regions of the plasma membrane were detected on the second representative NIH 3T3 cell (Fig. 2*F*, Kolmogorov-Smirnov test, *p* = 0.17) or the two other NIH 3T3 cells that we examined (*p* = 0.11 and 0.73, supplemental Table S1). Based on the lack of reproducible, statistically significant increases in ^18^O-enrichment at the ^15^N-sphingolipid domains on the NIH 3T3 and clone 15 cells, we conclude that the sphingolipid domains in the plasma membranes of fibroblast cells are not enriched with cholesterol.

##### Effect of mβCD Treatment on Cholesterol and Sphingolipid Distributions in the Membrane

**FIGURE 3.**
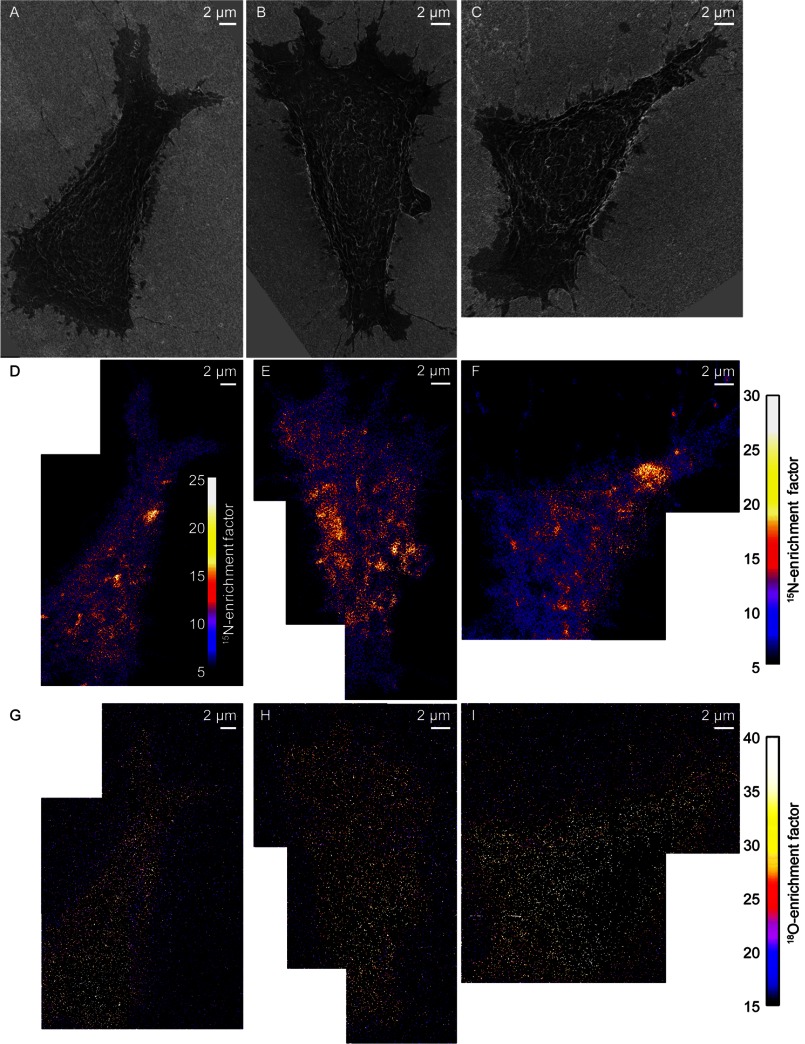
**SEM and SIMS images of cells that were treated with mβCD to reduce cellular cholesterol levels by 30%.** *A–C*, SEM images show the morphology of three metabolically labeled clone 15 cells that were treated with mβCD to reduce their cellular cholesterol levels. *D–F*, mosaics of ^15^N-enrichment SIMS images show mβCD treatment reduced the number of ^15^N-sphingolipid domains (*orange*, *yellow*, and *white* areas) in the plasma membranes of the clone 15 cells. The false color scale that quantifies the ^15^N-enrichment at each pixel is shown in the inset in *D*, and at the *right side* of the figure for *E* and *F. G–I*, mosaics of ^18^O-enrichment SIMS images of the clone 15 cells show the remaining ^18^O-cholesterol in the plasma membrane of each cell appeared to be relatively evenly distributed. Images of *B*, *C*, *E*, and *F* were adapted from Ref. 11.

**FIGURE 4.**
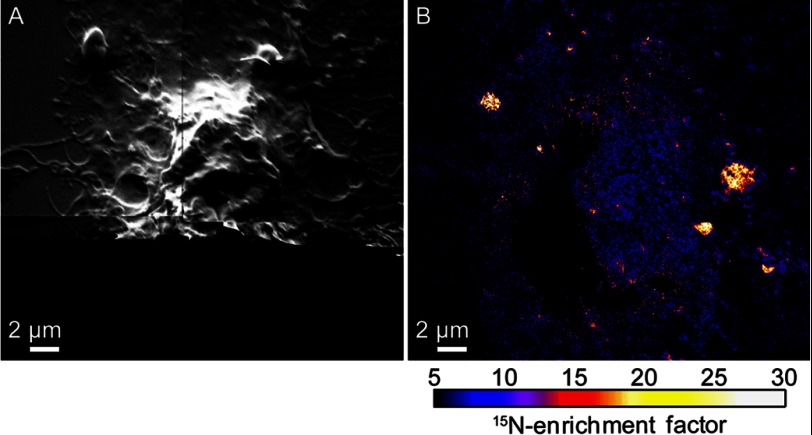
**High-resolution SIMS images of a NIH 3T3 cell treated with latrunculin A to disrupt its cytoskeleton.** *A*, montage of secondary electron images acquired with high-resolution SIMS shows latrunculin A treatment disrupted the cytoskeleton of the metabolically labeled NIH 3T3 cell. Secondary electrons were not detected near the *bottom* of the image due to the low primary ion beam current used for analysis. *B*, montage of ^15^N-enrichment SIMS images shows few ^15^N-sphingolipid domains were present in the plasma membrane of the NIH 3T3 cell after latrunculin A treatment. Latrunculin A treatment also disrupts the sphingolipid domains in the plasma membranes of clone 15 cells (11).

The lack of consistent ^18^O-cholesterol enrichment within the ^15^N-sphingolipid domains suggests that cohesive cholesterol-sphingolipid interactions are not responsible for the sphingolipid organization observed in the plasma membrane. And yet, acute depletion of cellular cholesterol with mβCD reduces the abundance and long range organization of the sphingolipid microdomains in the plasma membrane (11). To further investigate the potential role of cohesive cholesterol-sphingolipid interactions, we assessed whether mβCD treatment altered the cholesterol abundance within the sphingolipid domain and non-domain regions of the plasma membrane. Such changes in cholesterol distribution might occur if the composition of the plasma membrane prior to mβCD treatment was near a critical point (4), or if mβCD preferentially removed cholesterol from specific membrane domains (26). We reduced the cholesterol abundance in metabolically labeled clone 15 cells by ∼30% with mβCD. SEM imaging showed this level of cholesterol depletion may have slightly changed cell morphology and reduced the cell spreading area (Fig. 3, *A–C*), consistent with the known side effects of mβCD treatment (27–29). As we reported previously (11), mβCD treatment reduced the abundance of ^15^N-sphingolipid domains in the plasma membranes of the representative cells (Fig. 3, *D–F*), but to a lesser extent than that observed on cells whose cytoskeletons were disrupted with latrunculin A (Fig. 4, *A* and *B*). Although mβCD treatment reduced the amount of ^18^O-cholesterol on the cell surface, the remaining ^18^O-cholesterol in the plasma membrane was relatively uniformly distributed (Fig. 3*G*). No significant difference in the ^18^O-cholesterol abundance in the sphingolipid domain and non-domain regions was detected on the first (Fig. 3*G*) or second (Fig. 3*H*) representative mβCD-treated cell we examined (Kolmogorov-Smirnov test, *p* = 0.90 and 0.92, respectively, supplemental Table S1). However, Kolmogorov-Smirnov tests revealed a statistically significant reduction in ^18^O-cholesterol in the domains on a third mβCD-treated cell (Fig. 3*I*, *p* = 0.05) and a statistically significant increase in ^18^O-cholesterol in the sphingolipid domains on a fourth mβCD-treated cell (*p* = 0.04) (supplemental Table S1). Thus, mβCD did not reproducibly alter the cholesterol distribution in the plasma membrane or preferentially remove cholesterol from plasma membrane regions with distinct sphingolipid enrichment.

## DISCUSSION

### 

#### 

##### Sphingolipid Domains in the Plasma Membranes of Fibroblasts Are Not Lipid Rafts

Favorable interactions between cholesterol and sphingolipids are widely believed to induce the formation of domains enriched with these two components in the plasma membrane. However, no study involving directly imaging the distribution of cholesterol and sphingolipids in the plasma membrane without the use of potentially perturbing labels had been reported previously. The data presented in this study, which were acquired by detecting metabolically incorporated stable isotopes that do not alter the chemical composition of the lipids they label with high-resolution SIMS, indicate the sphingolipids and cholesterol are not co-localized in domains in the plasma membranes of fibroblast cells.

We had previously deduced that the sphingolipid domains were not lipid rafts due to their micrometer scale dimensions (lipid rafts are <200 nm) (3, 4) and their higher sensitivity to cytoskeleton disruption than cholesterol depletion (11). The lack of cholesterol enrichment in the sphingolipid domains, which is a critical characteristic of lipid rafts (3), definitively confirms this deduction. Moreover, our inability to detect cholesterol-enriched plasma membrane domains implies that if lipid rafts exist, they are either smaller than our lateral resolution (87 nm), or their abundance is insignificant relative to the more dominant sphingolipid-rich domains in the plasma membrane. This inference is consistent with magic angle spinning NMR spectral analysis of membranes even richer in hemagglutinin: the viral envelope, which shows an almost completely liquid disordered membrane at physiological temperature (14).

##### Cohesive Cholesterol-Sphingolipid Interactions Do Not Dictate Sphingolipid Organization in the Plasma Membranes of Fibroblasts

If cohesive interactions between cholesterol and sphingolipids play a significant role in organizing the plasma membrane, the sphingolipid-rich plasma membrane domains should be enriched with cholesterol. The lack of cholesterol enrichment in the sphingolipid domains, even after acute depletion of cellular cholesterol with mβCD, indicates that cohesive cholesterol-sphingolipid interactions do not induce sphingolipid domain formation in the plasma membranes of fibroblasts. Therefore, the reduction in sphingolipid domain abundance after mβCD treatment cannot be due to a loss of cohesive cholesterol-sphingolipid interactions. Instead, the effects of cholesterol depletion must be indirectly translated into changes in sphingolipid distribution through the involvement of other cellular components. Because our data show that disruption of the cytoskeleton eliminated the sphingolipid domains, and mβCD treatment is reported to alter cell morphology and spreading (27–29), the cytoskeleton appears to be involved in this process. Cholesterol-dependent scaffold proteins may also be involved because cholesterol binding to these scaffold proteins regulates the ability of the scaffold to interact with its target membrane proteins and form a functional signaling protein complex (30, 31). Overall, the dependence of the sphingolipid domains on an intact cytoskeleton supports a plasma membrane model in which lipid and protein organization is actively established by remodeling of the cortical actin (32, 33), and cholesterol plays a role in this process.

##### Implications for Other Types of Mammalian Cells

Here we tested the hypothesis that favorable sphingolipid-cholesterol interactions dictate sphingolipid organization in the plasma membrane (4) by using high-resolution SIMS to assess whether cholesterol was enriched in the sphingolipid domains in the membranes of fibroblast cells. Our investigation was restricted to two fibroblast cell lines because our previous study confirmed that the plasma membranes of these cells contained sphingolipid domains that were amenable to high-resolution SIMS analysis (11). Whether sphingolipids but not cholesterol are also organized into cytoskeleton-dependent domains in the plasma membranes of other cell types can only be inferred from this work. In the fibroblasts we studied, the sphingolipid-enriched plasma membrane domains were regulated by the cytoskeleton, and cohesive cholesterol-sphingolipid interactions were insufficient to drive the cholesterol to associate with the sphingolipid domains. Based on these findings, and the presence of a cytoskeleton in all mammalian cells, we anticipate that the plasma membranes of most types of mammalian cells contain sphingolipid domains that are not enriched with cholesterol, although the sizes of these domains may vary. To test this hypothesis, the metabolic labeling procedure would need to be optimized to selectively incorporate distinct stable isotopes into sphingolipids and cholesterol in more types of cells, and then the ^15^N-sphingolipid and ^18^O-cholesterol distributions in their plasma membranes would need to be imaged with high-resolution SIMS. Our current efforts include evaluating the plasma membrane organization in more diverse types of cells, as well as assessing the hypothesis that membrane traffic helps to modulate the sphingolipid organization in the plasma membrane (34–38). By supplementing fluorescence microscopy studies with complementary high-resolution SIMS imaging of isotope-labeled lipids in the plasma membrane, the mechanism that links lipid organization to the cytoskeleton and the role of cholesterol in this process may be elucidated.

## Footnotes

2 The abbreviations used are:

SIMS: secondary ion mass spectrometry
mβCD: methyl-β-cyclodextrin.
